# Lineage dynamics in growing biofilms: Spatial patterns of standing vs. *de novo* diversity

**DOI:** 10.3389/fmicb.2022.915095

**Published:** 2022-07-27

**Authors:** Ellen Young, Rosalind J. Allen

**Affiliations:** ^1^School of Physics and Astronomy, University of Edinburgh, Edinburgh, United Kingdom; ^2^Theoretical Microbial Ecology, Institute of Microbiology, Faculty of Biological Sciences, Friedrich Schiller University Jena, Jena, Germany

**Keywords:** biofilm, genetic diversity, lineage dynamics, evolution, spatial structure, agent-based simulation

## Abstract

Microbial biofilms show high phenotypic and genetic diversity, yet the mechanisms underlying diversity generation and maintenance remain unclear. Here, we investigate how spatial patterns of growth activity within a biofilm lead to spatial patterns of genetic diversity. Using individual-based computer simulations, we show that the active layer of growing cells at the biofilm interface controls the distribution of lineages within the biofilm, and therefore the patterns of standing and *de novo* diversity. Comparing biofilms of equal size, those with a thick active layer retain more standing diversity, while *de novo* diversity is more evenly distributed within the biofilm. In contrast, equal-sized biofilms with a thin active layer retain less standing diversity, and their *de novo* diversity is concentrated at the top of the biofilm, and in fewer lineages. In the context of antimicrobial resistance, biofilms with a thin active layer may be more prone to generate lineages with multiple resistance mutations, and to seed new resistant biofilms *via* sloughing of resistant cells from the upper layers. Our study reveals fundamental “baseline” mechanisms underlying the patterning of diversity within biofilms.

## 1. Introduction

Understanding how diversity is maintained within populations is one of the most important challenges in ecology and evolution (Barton and Keightley, 2002; Gibbons and Gilbert, 2015; Shade, 2017). Populations can adapt to changing environments *via* selection on pre-existing diversity (standing variation), and/or *via* selection on new (*de novo*) mutations, with different implications for the speed and nature of adaptation (Barrett and Schluter, 2008). The factors controlling the balance between standing and *de novo* diversity remain a topic of debate even for well-mixed populations (Barrett and Schluter, 2008). For spatially structured populations such as microbial biofilms the picture is more complex, since spatial structure can have drastic effects on evolutionary dynamics (Korona et al., 1994; Stewart and Franklin, 2008; Stacy et al., 2015).

Expanding populations are often characterized by genetic drift at the expanding front, leading to lineage loss and spatial segregation of surviving lineages (Habets et al., 2006; Hallatschek and Nelson, 2008, 2010; Perfeito et al., 2008; Excoffier et al., 2009; Nadell et al., 2010; Korolev et al., 2011; Freese et al., 2014; Mitri et al., 2016; Giometto et al., 2018). This has implications for the evolutionary maintenance of cooperative phenotypes (Ben-Jacob et al., 1994; Kreft, 2004; Habets et al., 2006; Bollback and Huelsenbeck, 2007; Park and Krug, 2007; Hallatschek and Nelson, 2008, 2010; Perfeito et al., 2008; Excoffier et al., 2009; Nadell et al., 2010, 2016; Korolev et al., 2011; Martens and Hallatschek, 2011; Mitri et al., 2011, 2016; Good et al., 2012; Mitri and Foster, 2013; Frost et al., 2018). In addition, some lineages that are located right at the growing front can expand dramatically, in a phenomenon known as gene surfing (Hallatschek et al., 2007; Hallatschek and Nelson, 2008, 2010; Gralka et al., 2016). Such spatial effects strongly influence the distribution of clone sizes for *de novo* mutations: bacterial colonies exhibit more jackpot events (large clones) compared to well-mixed populations (Fusco et al., 2016). Spatial effects can also lead to fragmentation of the population into independently evolving subpopulations (Fux et al., 2005; Steenackers et al., 2016). Moreover, evolutionary dynamics feeds back on the spatial structure of the population, for example through changes in growth speed or adhesive capacity (Kim et al., 2014; Steenackers et al., 2016; Kayser et al., 2018).

Microbial biofilms are widely observed to be phenotypically and genetically diverse (Hall-Stoodley et al., 2004; Stewart and Franklin, 2008; Stacy et al., 2015). This diversity is ecologically important, and probably contributes to the tolerance of clinical biofilms to antibiotic treatment (Mah and O'Toole, 2001; Stewart, 2002; Fux et al., 2005; Excoffier et al., 2009; Hallatschek and Nelson, 2010; Kim et al., 2014; Nadell et al., 2016; Frost et al., 2018). In environmental or clinical contexts, biofilms are likely to be seeded from genetically diverse inocula, such as skin, gut, soil, ocean, or river microbiota, so that standing variation may play a significant role. However, biofilms can also act as sources of *de novo* variation (Korona et al., 1994; Stewart and Franklin, 2008; Stacy et al., 2015). As we discuss below, spatial structure can drastically affect mutant fixation probabilities (Kim et al., 2014; Fusco et al., 2016). Spatial gradients of selection pressure, such as antibiotic, within the biofilm may also accelerate the emergence of resistant mutants, while the biofilm environment may favor the emergence of mutator strains and/or the horizontal transfer of genetic material (Stewart, 2002). In addition, spatial structure may promote the evolution of specific phenotypes that are well-adapted to the biofilm environment (Ben-Jacob et al., 1994; Nadell et al., 2010, 2016; Mitri et al., 2011; Mitri and Foster, 2013; Frost et al., 2018).

Biofilms are characterized by an uneven distribution of growth activity. Nutrients are rapidly consumed at the growing edge of the biofilm, so that the interior becomes nutrient-depleted. Therefore, growth is limited to a well-defined layer close to the biofilm front, where nutrient has not yet been consumed (Stewart and Franklin, 2008; Stacy et al., 2015; Stewart et al., 2016). This is known as the *active layer*; it has been observed in *in vitro* experiments (Pamp et al., 2008; Stewart et al., 2016) and in *ex vivo* clinical lab samples (Stewart et al., 2016), as well as in simulations (Xavier et al., 2004; Nadell et al., 2010, 2013; Young et al., 2022) and theory (Korolev et al., 2010). The width of the active layer is controlled by the balance between nutrient supply and consumption (Nadell et al., 2010). Hence, nutrient availability, nutrient consumption rate, nutrient diffusivity, biomass density and growth yield all affect the active layer width (Nadell et al., 2010). The active layer width is closely coupled to biofilm morphology: biofilms with thin active layers tend to have rough interfaces, while those with thick active layers tend to be smooth (Nadell et al., 2010; Farrell et al., 2013; Young et al., 2022)—although dynamical fluctuations of the active layer are also important (Young et al., 2022).

In this study, we investigate in detail how the spatial pattern of growth activity within biofilms leads to spatial patterns of standing and *de novo* diversity. Using individual-based biofilm simulations, we track the fate of hundreds of neutral cell lineages in growing biofilms. Our simulations allow direct observation of the loss of standing diversity, and we infer the gain of *de novo* diversity from patterns of lineage length. In this work, we choose to compare biofilms grown to equal *size*, under conditions where the active layer thickness is different. Our study complements previous work by Mitri et al. (2016), who studied diversity in bacterial colonies, grown for equal *time* with differing nutrient availability. Increasing nutrient availability increases the active layer width (Nadell et al., 2010). Mitri et al. (2016) observed that well-fed colonies retain standing diversity over more generations than poorly fed colonies; however over a similar timescale, well-fed colonies undergo more generations of growth than poorly-fed ones. Therefore, comparing colonies over the same timescale, well-fed and poorly-fed colonies retain similar amounts of standing diversity since the differences in colony size compensate for the differences in active layer thickness. Here, our aim is to understand the fundamental role of the active layer, for which the picture is clearer when we compare biofilms of equal size.

We find that active layer thickness controls both the balance between standing and *de novo* variation, and the spatial patterns of *de novo* mutations within the biofilm. For biofilms of equal size, those with a thick active layer retain more standing diversity and their *de novo* diversity is more evenly distributed across the biofilm. In contrast, biofilms with a thin active layer retain less standing diversity, and their *de novo* diversity is concentrated close to the growing interface. Since *de novo* diversity is concentrated in fewer lineages, the occurrence of multiple mutations along the pathway to high-level antibiotic resistance is more likely in biofilms with thinner active layers. In this study, we do not aim to represent biofilm growth and evolution in realistic detail, but rather to provide a baseline model that reveals fundamental mechanisms connecting spatial patterning of growth and diversity, onto which more complex effects can be superposed.

## 2. Methods

### 2.1. Agent-based simulation algorithm

In this work, we use the individual-based biofilm modeling software iDynoMiCS (Lardon et al., 2011). iDynoMiCS models the microbes in a biofilm as individual agents whose behavior is coupled to a nutrient reaction-diffusion equation (Lardon et al., 2011). The agents, which in this work are assumed to be discs in continuum 2D space, grow with specific growth rate μ according to the Monod equation μ = μ_*max*_*S*/(*k*_*S*_+*S*), where μ_*max*_ is the maximum specific growth rate, *k*_*S*_ is the concentration of nutrient at which the growth is half maximal, and *S* is the local nutrient concentration at the position of the microbial agent (Monod, 1949). Once a microbial agent reaches a maximum radius (which has a stochastic element), it divides into two daughters. Microbes interact with one another mechanically *via* a shoving algorithm. Briefly, this algorithm detects pairs of agents whose “zones of influence” (defined to be the radius multipled by a “shove parameter”) overlap, and shuffles the agent positions to avoid such overlaps (Lardon et al., 2011). Although iDynoMiCS has the facility to model extra-cellular matrix (EPS) as non-replicating particles, we did not model EPS in this study.

In iDynoMiCS, the computational domain is set up to resemble a flow cell, in which the biofilm grows on a hard surface and nutrients diffuse from above. The nutrient is represented by a concentration field which varies in space and time due to diffusion and consumption by the microbes. A separation of timescales is assumed, such that the reaction-diffusion equation for the nutrient is assumed to reach steady state faster than the timescale for microbial growth; hence the reaction-diffusion equation for the nutrient concentration is solved to steady state at each iteration of the microbial growth algorithm. Convective flow is not modeled, but rather it is assumed that there is a stationary layer of fluid close to the biofilm: the “boundary layer” (Kreft et al., 2001; Lardon et al., 2011). It is also assumed that the diffusion constant for nutrient is reduced inside the biofilm by a fixed factor compared to outside the biofilm. The input values used in our simulations are based on experimental values for oxygen-limited *Pseudomonas aeruginosa* biofilms (see Table 1). We vary the bulk nutrient concentration (*S*_*bulk*_) and the maximum specific growth rate (μ_*max*_) in order to simulate biofilms with different spatial structures. They could in principle be controlled experimentally by changing the nutrient concentration of the fluid medium in a flow cell setup, and the bacterial strain.

**Table 1:** Input values used in our iDynoMiCS biofilm simulations.

**Parameter**	**Values**	**Description**	**References**
*S*_*bulk*_	10^−3^−10^−2^ g/L	Bulk concentration of limiting nutrient (here assumed to be oxygen). This value is varied to alter biofilm morphology.	Saturation concentration of water at 37^*o*^C is 6.6 × 10^−3^ g/L (Battino et al., 1983)
*Y*	0.64 g/g	Yield—grams of biomass produced per gram of oxygen consumed	(Beyenal et al., 2003)
μ_*max*_	0.1-0.4 /h	Maximum specific growth rate. This value is varied to alter biofilm morphology	(Bakke et al., 1984; Robinson et al., 1984; Beyenal et al., 2003; Kragh et al., 2016)
*k*_*S*_	8.12 × 10^−4^ g/L	Concentration of oxygen at which the growth is half maximal	(Kragh et al., 2016)
*D*_*S*_	2.3 × 10^−4^m^2^/day	Diffusion coefficient of nutrient (oxygen)	(Stewart, 2003)
Biofilm diffusivity	0.8	Factor multiplying *D*_*S*_ to give nutrient diffusion coefficient inside the biofilm	(Rittmann and Manem, 1992; Stewart, 2003)
*h*	80 μm	Diffusion boundary layer height	(Picioreanu et al., 1998; Xavier et al., 2005; Alpkvist et al., 2006)
ρ_*B*_	200 g/L	Biomass density of microbes in biofilm	(Xavier et al., 2005; Bjarnsholt et al., 2009)
*r*_*div*_	2 μm	Average radius of microbial agent at division	(Beyenal et al., 2003)
*k*_*Shov*_	1.15	Factor multiplying the agent's radius to give the shove radius	Default iDynoMiCS value (Lardon et al., 2011)
*L*_*y*_	1,032 μm	Width of the simulation domain	
*N*_0_	300	Number of initialized microbial agents	

*These values are loosely based on Pseudomonas aeruginosa in a flow cell type set up (Melaugh et al., 2016)*.

To be able to simulate biofilm growth over long times, we use a “clipping” algorithm in combination with iDynoMiCS (Young et al., 2022). This algorithm periodically removes inactive agents far below the growing front, such that a computationally feasible number of agents remain in the simulation space. This is achieved by pausing the iDynoMiCS simulation and removing the relevant agents, or clipping, and then restarting the simulation. This clipping procedure is done at regular time intervals. In the clipping procedure, microbial agents which are located both below the lowest actively growing agent and below the minimum point of the interface (which can be different points depending on the biofilm configuration), are removed. The complete algorithm has been described by Young et al. (2022).

### 2.2. Tracking microbial lineages

To study the microbial lineages in our simulations, we use built-in iDynoMiCS variables that relate to the genetic tree, namely the family number and the generation number (Lardon et al., 2011). The family number (1…*N*_0_) labels the descendants of each of the *N*_0_ agents that were present at the start of the simulation. Upon a division event, both daughter agents inherit the family number of the parent. The generation number allows us to measure the lineage lengths of the agents, i.e., the number of divisions that have happened in the lineage of that agent since the start of the simulation. The generation number is set to zero for all agents at the start of the simulation. Upon a division event, both daughters are assigned a generation number which is greater by 1 than the generation number of the parent.

### 2.3. Defining and measuring the active layer

We define the active layer as the layer of growing microbial agents at the top of the biofilm. More specifically, we define a threshold growth rate; agents which grow faster than this rate are defined to be part of the active layer. We consider an agent to be in the active layer if its growth rate is >0.1% of the maximal growth rate μ_*max*_*S*_*bulk*_/(*k*_*S*_+*S*_*bulk*_) that is possible under the conditions of the simulation (i.e., for the chosen values of μ_*max*_ and *S*_*bulk*_). Therefore, the condition for an agent to be part of the active layer is μ>(1/1000) × μ_*max*_*S*_*bulk*_/(*k*_*s*_+*S*_*bulk*_).

To calculate the average active layer thickness we define a grid spanning the simulation domain with *D* columns (horizontal bins) and *H* rows (vertical bins) of width 8μm. Within each of the *D* columns, we find the total number of “active” grid squares whose biomass has an average specific growth rate above the active layer threshold. The local active layer thickness is then the number of active grid squares within the column, multiplied by the 8μm height of a grid square. We note that for some biofilm configurations, for example if the biofilm is rough, the active grid squares within one column may not necessarily be adjacent to one another. Once the local active layer thickness for each vertical column has been found, the mean active layer thickness across the biofilm is found by averaging these values over all the *D* columns.

## 3. Results

### 3.1. Agent-based simulations show diverse biofilm morphology and active layer structure

We used agent-based simulations with iDynoMiCS (Lardon et al., 2011) to model the growth of microbial biofilms over long times, starting from an initial population of 300 “founder” microbes. Our simulations model individual microbes as disc-shaped agents which consume nutrients, grow, divide, and push each other out of the way (see section Methods). Our model is neutral, in the sense that all microbes are, *a priori*, equally fit. To focus on spatial patterns of growth and diversity, without confounding effects of biofilm size, we compare biofilms grown to equal size, for different parameter values.

We observe different biofilm morphologies for different parameter values, consistent with previous work (Xavier et al., 2004; Korolev et al., 2010; Nadell et al., 2010; Stacy et al., 2015; Young et al., 2022) (Supplementary Figure 1; see also the Supplementary Movies). For high nutrient concentration or low values of the microbial maximal growth rate parameter μ_*max*_ the biofilm interface is smooth, while for low nutrient concentration or high μ_*max*_ it becomes fingered (Supplementary Figure 1). We designate individual microbes as “active” if their growth rate exceeds a threshold of 0.1% of the maximum growth rate achieveable in the simulation (see section Methods).

**Figure 1:**
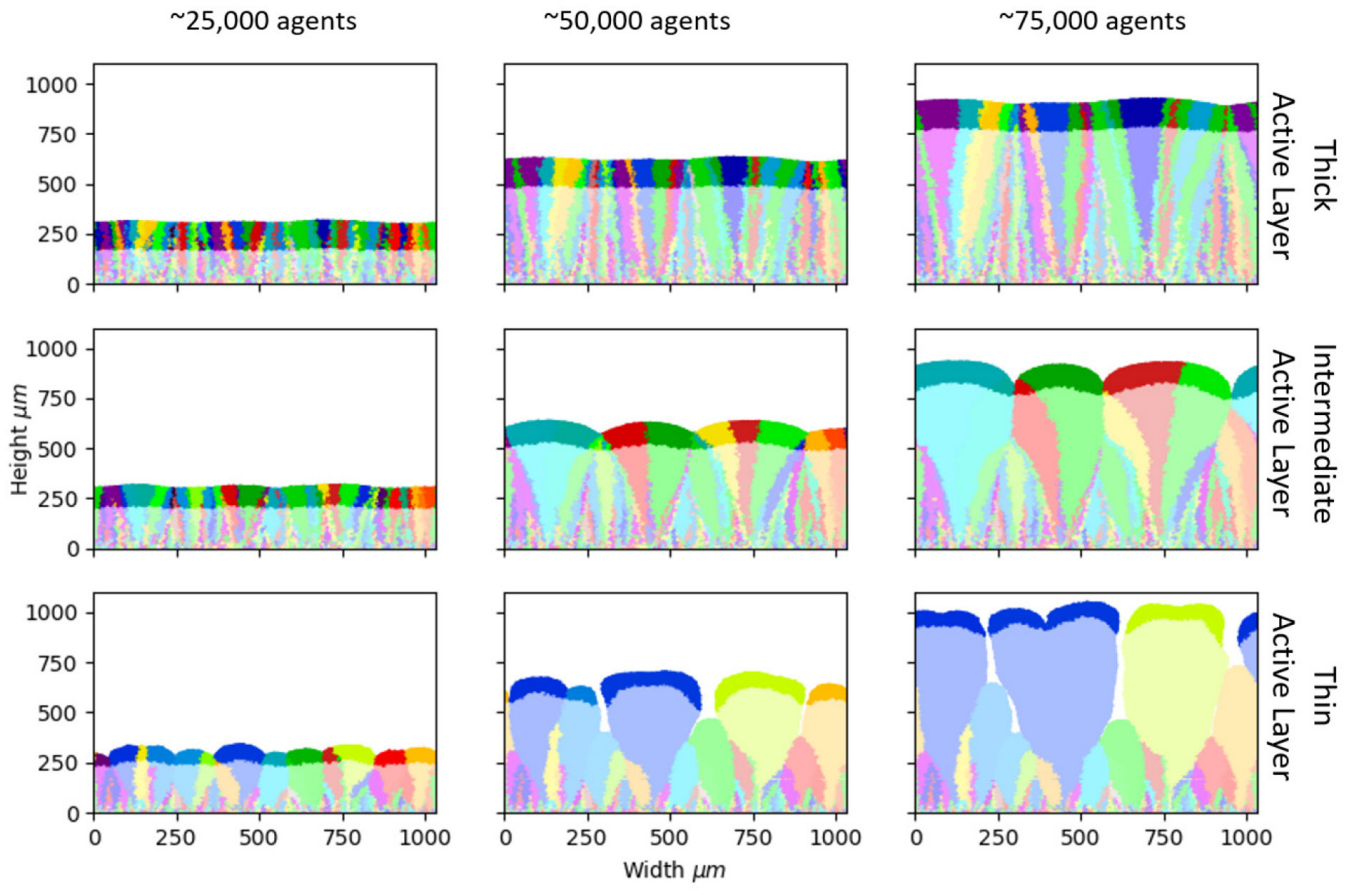
Biofilm morphology and loss of standing diversity. Snapshots from our simulations at different stages of biofilm growth (left to right: 25,000, 50,000, and 75,000 microbial agents). The active layer is shown by the shaded region (see section Methods for definition). Three simulations are shown (**top, middle**, and **bottom** rows), with different parameters and hence different values of the active layer thickness. **(Top)** *S*_*bulk*_ = 0.01 g/L; μ_*max*_ = 0.1/h; producing an average active layer thickness of 102.8±0.8 μm. **(Middle)** *S*_*bulk*_ = 0.005 g/L; μ_*max*_ = 0.2/h; average active layer thickness 71.3±1.4 μm. **(Bottom)** *S*_*bulk*_ = 0.001 g/L; μ_*max*_ = 0.3/h; average active layer thickness 40.8±1.4 μm. The rest of the simulation parameters are as in Table 1. The descendants of each of the 300 founder cells are shown in a different color, allowing visualization of the patterns of loss of standing diversity.

As expected, active microbes are located in a layer close to the biofilm interface (colored region in Supplementary Figure 1; shaded region in Figures 1, **4**). Tracking the average thickness of this active layer across the biofilm interface (see section Methods), we find that it stabilizes early in biofilm growth (Supplementary Figure 2). High nutrient concentration, or low values of the maximal growth rate μ_*max*_, lead to a thick, continuous, active layer while low nutrient concentration or high μ_*max*_ lead to a thin active layer that has gaps, corresponding to the troughs between the biofilm fingers (Supplementary Figures 1, 2 and Supplementary Table 1; Young et al., 2022). For intermediate nutrient concentration or μ_*max*_ the active layer is of intermediate thickness and is dynamic, with transient gaps appearing and disappearing (see the kymograph in **Figure 3**; Young et al., 2022).

### 3.2. Active layer thickness controls loss of standing diversity *via* genetic drift

We first investigate the loss of standing diversity during biofilm growth. We label each of the 300 founder cells with a different “color” that is inherited upon division, allowing us to track the founder cell's descendants (see section Methods). The colors in Figure 1 illustrate the fates of the 300 founder cell lineages, for three simulations with different active layer thickness. In all simulations, genetic drift leads to loss of standing diversity, such that the active layer becomes dominated by just a few founder lineages (Figure 1).

However, the loss of standing diversity proceeds very differently in our three simulations. Comparing biofilms of equal size, more standing diversity is lost from the biofilm with the thinner active layer (bottom row in Figure 1), while less standing diversity is lost from the biofilm with a thicker active layer (top row in Figure 1).

**Figure 2:**
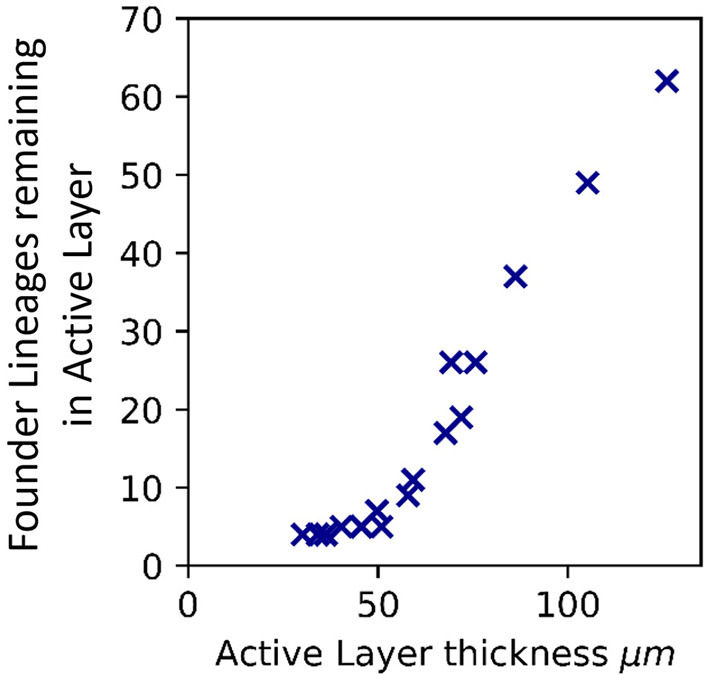
Active layer thickness controls loss of standing diversity. Correlation between the number of founder lineages remaining in the active layer, and thickness of the active layer (averaged across the biofilm interface), for 16 simulated biofilms of size 50,000 microbial agents. The active layer thickness was varied by changing the bulk nutrient concentration (*S*_*bulk*_) and the maximum specific growth rate (μ_*max*_). The values of *S*_*bulk*_ and μ_*max*_ corresponding to these simulations are shown in Supplementary Table 1 together with the active layer thicknesses. The rest of the simulation input parameters are as in Table 1. Supplementary Figure 3 shows the same plot for biofilms that have reached 25,000, 75,000, and 100,000 microbial agents.

To probe the link between active layer thickness and loss of standing diversity, we performed more simulations to generate biofilms with a wide range of active layer thicknesses (Supplementary Table 1). We counted the number of founder lineages that remained in the active layer at a biofilm size of 50,000 microbes: this provides a quantitative measure of the retention of standing diversity. Retention of standing diversity is strongly correlated with the active layer thickness (Figure 2 and Supplementary Figure 3). Comparing biofilms of equal size, those with thicker active layers have larger effective population size and are less subject to genetic drift, so they retain more standing diversity.

### 3.3. Active layer dynamics causes local losses of standing diversity

We hypothesized that loss of standing diversity might depend not just on the average active layer thickness but also on the local dynamics of the active layer. Across the biofilm interface, the local active layer thickness can vary quite dramatically (Young et al., 2022; Supplementary Figures 1, 2). For example, our simulation with intermediate active layer thickness shows transient gaps in the active layer, corresponding to troughs between bulges in the interface (Figure 1 and Supplementary Figure 1). In previous work, we have shown that these gaps cause pinning of the interface, leading to a rough morphology (Young et al., 2022).

**Figure 3:**
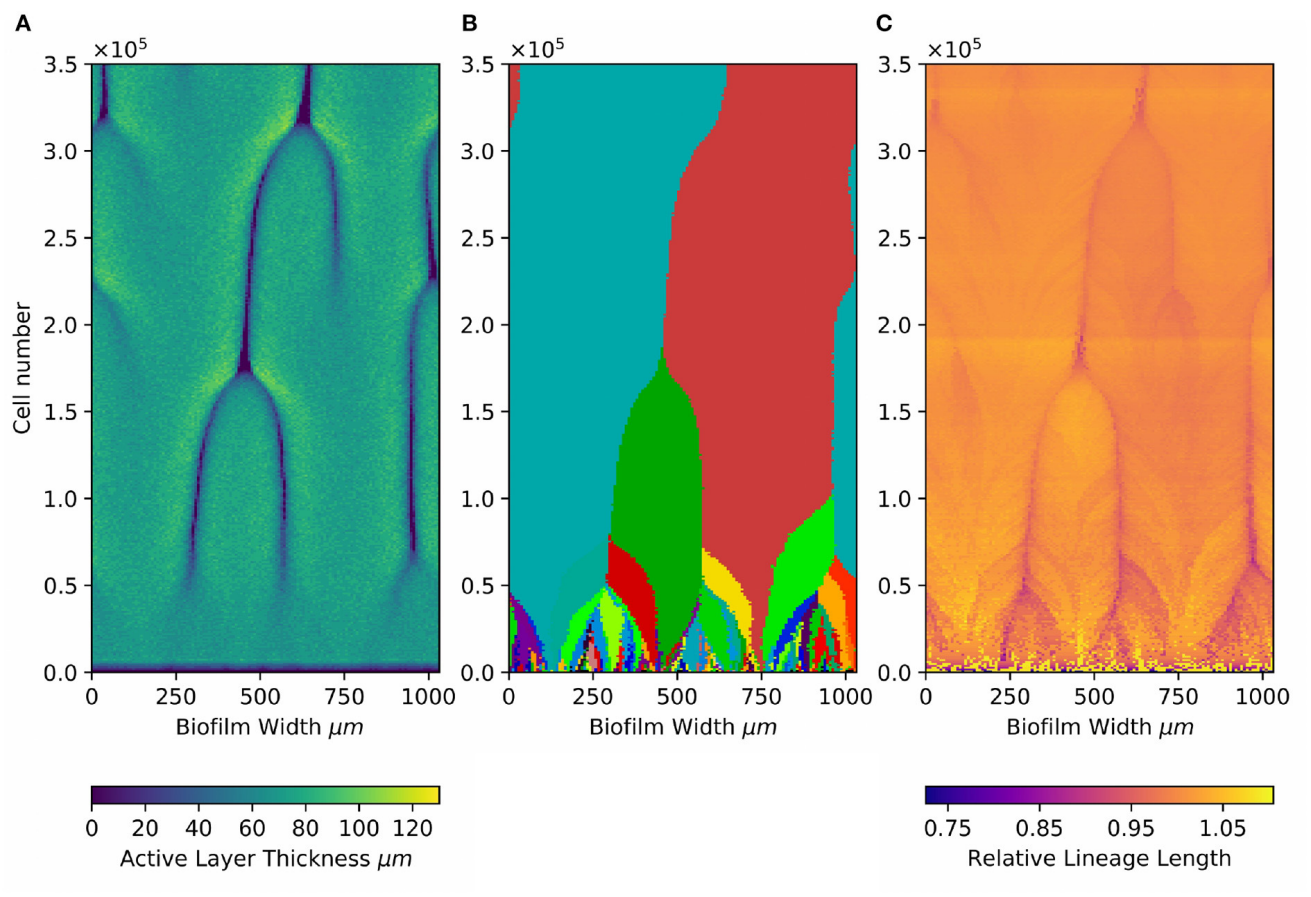
Local active layer dynamics affects both loss of standing diversity and patterns of *de novo* diversity. Kymographs showing **(A)** dynamical changes in the active layer, **(B)** dynamics of the 300 founder lineages, and **(C)** dynamics of the relative lineage length at different positions along the biofilm interface. Results are shown for the simulation at intermediate active layer thickness [71.30±1.42 μ*m* (*S*_*bulk*_ = 0.005*g*/*L*; μ_*max*_ = 0.2; middle row in Figures 1, 4)], In this simulation, the active layer shows transient gaps (Young et al., 2022). **(A)** Shows how the local active layer thickness (colorscale) across the width of the biofilm (horizontal axis) changes during biofilm growth (vertical axis show the total number of agents in the biofilm, as a proxy for time). The darker lines correspond to the movement of local gaps in the active layer. The merger of two dark lines happens when a bulge in the biofilm interface is subsumed by two adjacent bulges and is lost behind the growing front (Young et al., 2022). **(B)** Shows the founder cell lineages present at the biofilm interface. Lineages of the 300 founder microbes are indicated using the same colors as in Figure 1. **(C)** Shows the dynamics of the relative lineage length (color scale) for microbes located at the interface. The relative lineage length is calculated as the lineage length of an individual microbe located at the interface, divided by the average lineage length of all the microbes located at the interface at that time point. Plotting the relative lineage length makes it easier to see local trends which would be obscured by the much larger general increase in lineage length with time as the biofilm grows (Figure 4).

Our simulations show that founder lineages tend to be lost at local sites where there are active layer gaps. To observe this, we plot an “active layer kymograph” for the simulation at intermediate nutrient concentration (Figure 3A). Here, the colors represent the local active layer thickness along the biofilm interface (horizontal axis), with biofilm size being shown on the vertical axis (Young et al., 2022). Local gaps in the active layer appear as dark lines, whose dynamics can be observed by reading from bottom to top. The merger of two active layer gaps corresponds to an event where a bulge in the interface is subsumed by two adjacent larger bulges (Young et al., 2022).

To correlate loss of standing diversity with active layer dynamics, we also make a kymograph for the dynamics of the 300 founder lineages in the same simulation (Figure 3B). To make this plot, we record in the horizontal direction the founder ancestor of every microbial agent along the biofilm interface (using the same colors as in Figure 1), and juxtapose data for different biofilm sizes along the vertical axis. This allows us to visualize the dynamics of loss of founder lineages as the biofilm grows (bottom to top in Figure 3B). Eventually, only 2 founder lineages remain.

Comparing the active layer dynamics with the founder lineage dynamics (Figures 3A,B) shows a clear correlation. Local losses of founder lineages happen when active layer gaps merge, i.e., when local bulges in the biofilm interface become subsumed behind the growing front. When this happens, all founder lineages that are located within the subsumed bulge are lost. Therefore, local active layer dynamics can produce hot spots for loss of standing diversity. This suggests that both local active layer dynamics and the average thickness of the active layer are relevant factors controlling the loss of standing diversity as the biofilm grows.

### 3.4. Active layer thickness controls distribution of *de novo* genetic diversity in space and among lineages

Next, we investigate how *de novo* diversity is affected by active layer thickness. Our simulations do not model mutation events directly. However, in our neutral model, mutations can be assumed to occur with equal probability at each division event. The number of mutations that a lineage accumulates is expected to be proportional to the number of divisions in that lineage, going back to the founder cell—in other words, the lineage length (see section Methods). Our simulations allow us to track the lineage length of every microbial agent within the biofilm, and therefore to infer the number of (neutral) mutations that are expected to have accumulated.

In this work, we compare biofilms of equal size. Therefore each biofilm has undergone the same number of divisions and is expected to contain the same total *de novo* diversity (number of mutations). However, the spatial patterning of *de novo* diversity within the biofilm, and its distribution among lineages, may be different.

**Figure 4:**
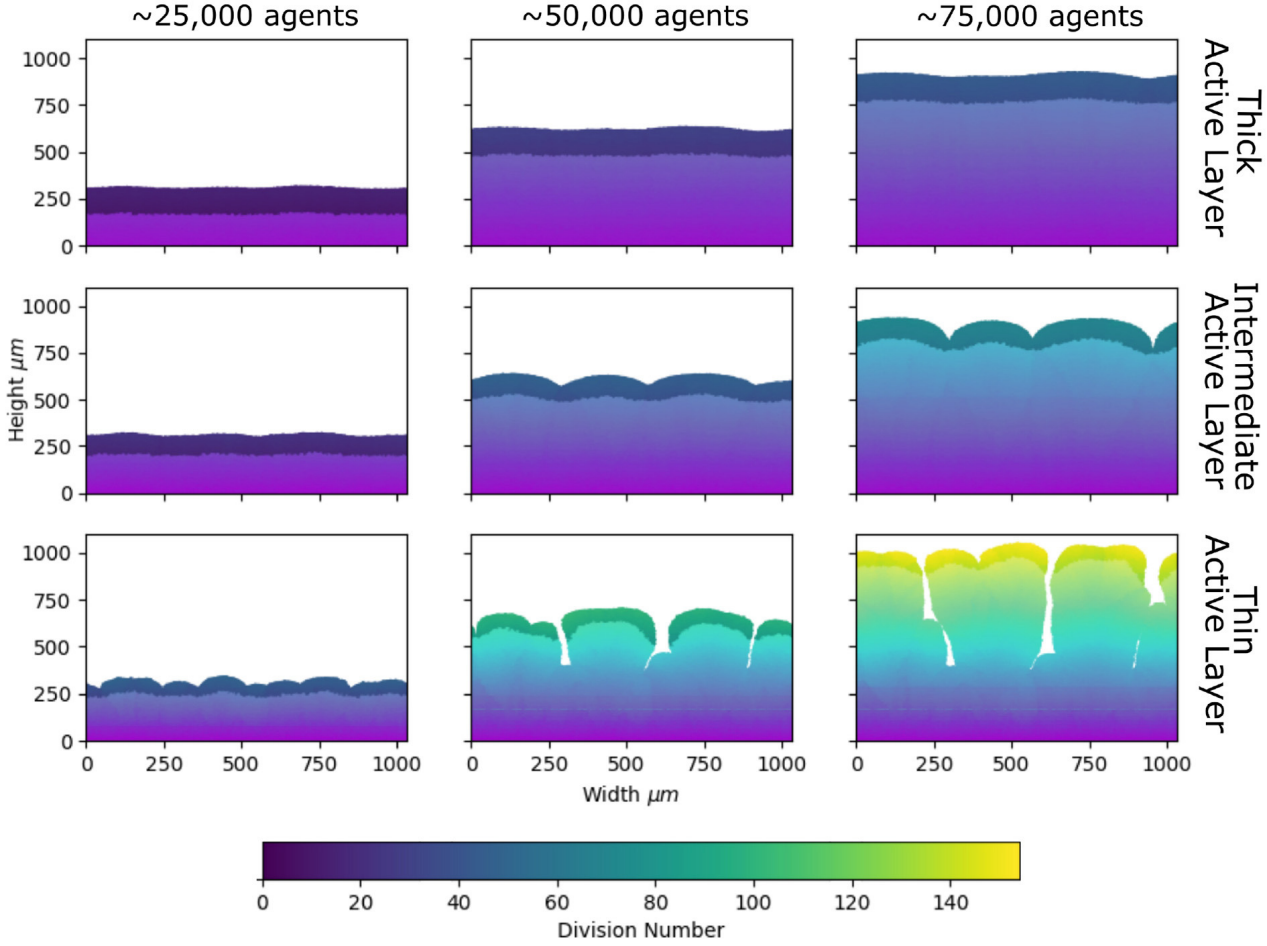
Patterns of *de novo* diversity, inferred from lineage length. Snapshots from our simulations at different stages of biofilm growth, as in Figure 1, but color-coded according to lineage length (left to right: 25,000, 50,000, and 75,000 microbial agents; top to bottom: average active layer thicknesses 102.8±0.8, 71.3±1.4, and 40.8±1.4 μ*m*; parameters are given in the caption of Figure 1 and Table 1). Agents are colored according to their lineage length, i.e., the number of divisions that have occurred in the history of that agent since the start of the simulation (see Section Methods). The region of darker shading indicates the active layer (see section Methods).

Mapping the spatial distribution of lineage length in our simulated biofilms, we observe clear patterns (Figure 4 and Supplementary Movies). In all our simulations, lineage length increases linearly with vertical height within the biofilm (Supplementary Figure 4). This happens because lineages are terminated when they fall behind the growing front (Schreck et al., 2019); the trend is linear because the biofilm grows linearly in time. Since longer lineages accumulate more mutations, our results imply that mutations will be concentrated preferentially in the upper parts of a growing biofilm. This is relevant, because mutations in the upper parts of the biofilm are more likely to propagate as the biofilm grows, and also have more chance of spreading if cells detach from the biofilm and go on to seed new biofilms.

Comparing our simulations for high, intermediate and low active layer thickness (Figure 4), we see clear differences in the spatial pattern of lineage lengths. In the biofilm with the thinner active layer, lineage length varies more across the biofilm, whereas it is more homogeneous in the biofilm with the thicker active layer (Figure 4). This implies that, comparing biofilms of equal size, mutations will be more strongly concentrated at the growing edge if the biofilm has a thin active layer, and more evenly spread across the biofilm if the active layer is thick.

**Figure 5:**
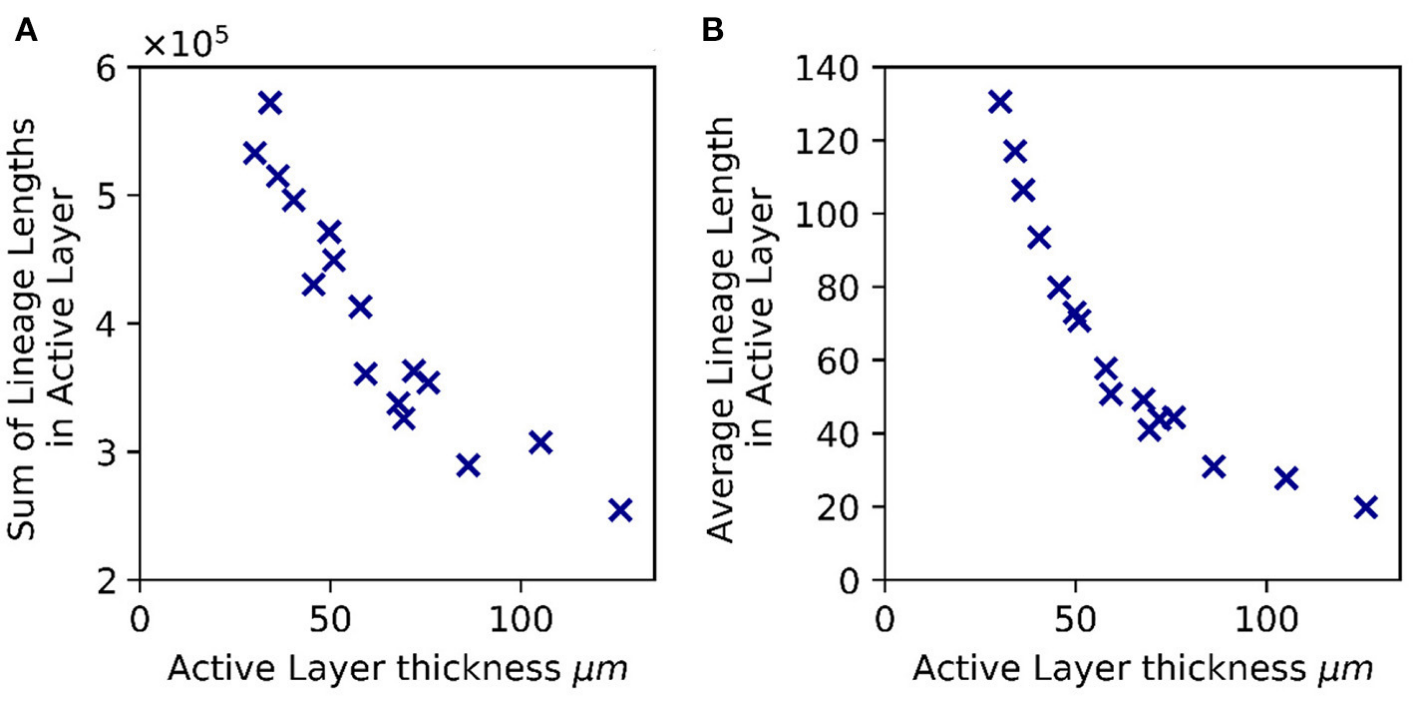
Active layer thickness controls patterns of lineage length, hence *de novo* diversity. **(A)** Total *de novo* diversity in the active layer. The sum of the lineage lengths of all microbial agents in the active layer is plotted against the active layer thickness (averaged across the biofilm interface) for 16 biofilms that have reached 50,000 agents. **(B)** Average lineage length of a microbial agent in the active layer, plotted vs. the active layer thickness. In both panels, as in Figure 2, the active layer thickness was varied by changing the bulk nutrient concentration (*S*_*bulk*_) and the maximum specific growth rate (μ_*max*_). The values of *S*_*bulk*_ and μ_*max*_ corresponding to these simulations are shown in Supplementary Table 1 together with the active layer thicknesses. The rest of the simulation parameters are as in Table 1.

To further investigate the link between active layer thickness and spatial patterning of mutations, we re-analyzed our more extensive set of simulations with a broad range of active layer thicknesses (Supplementary Table 1). Since we compare biofilms of equal size we would expect (on average) the same total number of mutations for all these biofilms. However, mutations may be differently distributed within the biofilm. To estimate the extent to which mutations concentrate at the top of the biofilm, we computed the sum of lineage lengths for all microbes *in the active layer*. This quantity correlates strongly with the active layer thickness (Figure 5A and Supplementary Figure 5). Therefore, in biofilms with a thinner active layer, we expect mutations to be concentrated at the top of the biofilm, within the active layer, while for biofilms with a thicker active layer, we expect mutations to be more widely distributed, occurring within the dormant lower layers of the biofilm as well as within the active layer.

High-level resistance to antibiotics often requires multiple sequential mutations (Toprak et al., 2011; Greulich et al., 2012). Long lineages are more likely to accumulate multiple resistance mutations. To estimate the propensity for biofilms to gain high-level antibiotic resistance, we computed the average lineage length for individual microbes in the active layer, for our simulation set. This quantity also correlates strongly with the active layer thickness (Figure 5B and Supplementary Figure 5). This suggests that, *a priori*, biofilms with a thin active layer are more prone to *de novo* evolution of high-level resistance, compared to biofilms of the same size with a thicker active layer.

How does active layer thickness control the patterning of *de novo* genetic diversity within a biofilm? Put simply, replication events are confined to the active layer (i.e., the active layer thickness determines the effective population size). If the active population is of size *N*_*act*_ and the biofilm contains *N*_*tot*_ microbes in total, then the average lineage length of microbes in the active population must be *N*_*tot*_/*N*_*act*_. Biofilms with a thin active layer have small *N*_*act*_ and therefore long lineages for microbes at the biofilm interface. In contrast, biofilms with a thicker active layer have larger *N*_*act*_ and the lineage length at the interface is correspondingly shorter.

Our simulations also show that the local active layer dynamics affects spatial patterns of lineage length. Figure 3C illustrates with a kymograph the local dynamics of lineage length at the biofilm interface, during biofilm growth. Here, the color scale shows the lineage length for microbes along the biofilm interface, relative to the average lineage length for microbes at the interface. The horizontal axis indicates position along the biofilm interface, while the vertical axis indicates cell number. Lighter colors show local regions of greater than average lineage length, which are predicted to be local hot spots, where mutations are more likely to be found. Comparing the pattern of lineage length (Figure 3C) to that of active layer thickness (Figure 3A) shows that lineage length is locally longer where the active layer is locally thicker, in other words, at the peaks of bulges along the biofilm interface. However, this local effect is minor compared to the effect of the average active layer thickness.

## 4. Discussion

Biofilms often show high levels of genetic diversity, which is believed to contribute to antibiotic tolerance and resistance (Mah and O'Toole, 2001; Stewart, 2002). Understanding whether this diversity primarily arises from pre-existing (standing) variation or from newly generated (*de novo*) variation has significant implications. For example, adaptation to environmental challenges is generally faster from a basis of standing variation (Barrett and Schluter, 2008). Here, we used an individual-based biofilm model, to show how the spatial patterns of microbial growth within a biofilm lead to spatial patterns of standing and *de novo* diversity. Our work reveals a central role for the active layer of growing microbes at the biofilm interface. Comparing biofilms of equal size, a biofilm with a thick active layer retains more standing diversity, and its *de novo* diversity is more evenly distributed, both spatially and among individuals in the population. In contrast, a biofilm with a thin active layer retains less standing diversity, and its *de novo* diversity is concentrated close to the biofilm interface, with relatively less *de novo* diversity being located in the deeper parts of the biofilm. This implies that microbes with multiple mutations, leading to high-level antibiotic resistance, are more likely in biofilms with a thin active layer, compared to biofilms of equal size with a thick active layer. We also find that the local dynamics of the active layer plays a role, for example, causing local hot spots of loss of standing variation when interface bulges are lost behind the growing front.

Putting our results together, our model predicts contrasting spatial patterns of standing diversity and *de novo* diversity. Standing diversity is greatest in the lower parts of the biofilm, while *de novo* diversity is greatest at the top of the biofilm. This could have consequences when biofilms are subjected to environmental challenges. For example, antibiotics that target primarily the active, upper, part of the biofilm would tend to select on *de novo* diversity, while those that target primarily the lower part of the biofilm might select on standing diversity (Pamp et al., 2008). Likewise, sloughing of the upper layers of a biofilm might disperse *de novo* diversity to the wider environment, while leaving standing diversity in place.

In this work, we compared biofilms grown to equal *size*, with different active layer thickness, achieved by varying the parameters of our individual-based model. In doing this, we follow the work of Drescher et al. (2016), who also point to biofilm size, rather than age, as a key control parameter. This contrasts with the work of Mitri et al. (2016), who compared bacterial colonies grown for equal *time*, on media with varying nutrient availability. Mitri et al. (2016) found that, overall, nutrient availability had little effect on loss of standing diversity, because the differences in colony size counteracted the effects of the active layer thickness. In this work, we aimed to elucidate the fundamental mechanisms by which growth patterning leads to patterning of diversity. These mechanisms are clearer when we compare biofilms of equal size. One might argue that comparing biofilms of equal size restricts the practical relevance of our conclusions, since slow-growing biofilms will generally be smaller than fast-growing ones. However, in the natural environment, biofilm maturity does not necessarily correspond to increasing size: biofilm growth can be limited by space (e.g., inside a cavity in a medical implant) or by chemical interactions (e.g., the secretion of pulcherrimin which causes growth arrest in *Bacillus subtilis* colonies; Arnaouteli et al., 2019). Bearing in mind that our comparison is made for biofilms of equal size, it would be important to carefully define the conditions for any experimental test of these predictions.

To control the active layer thickness in our simulations, we varied two model parameters: the bulk nutrient concentration *S*_*bulk*_ and the maximal specific growth rate μ_*max*_. We could have chosen to vary a single parameter. For example, increasing *S*_*bulk*_ alone (as in the study of Mitri et al., 2016) increases the active layer thickness, but it also increases the average activity of microbes within the active layer (Supplementary Figure 6 and Supplementary Table 1). Increasing μ_*max*_ alone decreases the active layer thickness, while increasing the average activity of microbes within the active layer (Supplementary Figure 6 and Supplementary Table 1). By varying multiple parameters, we can identify the active layer thickness as the controlling factor, rather than other factors, such as the activity of individual microbes, that correlate with individual parameters.

Importantly, we have assumed neutrality in this study: *a priori*, all microbial agents in our simulations have equal fitness and identical traits. This allows us to predict patterns of mutations within the biofilm from lineage lengths, without explicitly simulating mutation events. Neutral models have a distinguished history in ecology and evolution (Volkov et al., 2003; Azaele et al., 2006); they are useful for predicting baseline phenomena, deviations from which can point to specific biological mechanisms. In this study, the predicted baseline phenomenon is the connection between the active layer and patterns of standing and *de novo* diversity. Neutral models do not provide a realistic description of the real biological system, but they do provide a useful reference to which to compare biological measurements (Nee, 2005).

Similarly, our study aims to elucidate baseline mechanisms, rather than to provide a realistic model for an evolving biofilm. Our model neglects many biological and physical factors, including fitness effects of mutations, antibiotic effects on mutation rates, the emergence of hypermutators, persisters, physical effects of exopolysaccharide production, 3D geometric effects and fluid flow. All of these could produce different outcomes for the patterning of standing and *de novo* diversity within a biofilm, and should be investigated in future work. Feedback between evolutionary processes and the spatial structure of the population (e.g., the formation of biofilm bulges by fitter mutant clones, or a change in the local active layer thickness due to a mutant with a different growth yield) could also have interesting effects.

Previous work on evolution in spatially expanding microbial populations has focused on the distribution of clone sizes, i.e., the number of descendants of a mutant that emerges within the population (Hallatschek et al., 2007; Hallatschek and Nelson, 2008, 2010; Fusco et al., 2016; Gralka et al., 2016; Farrell et al., 2017; Schreck et al., 2019). The clone size distribution is different in a spatially expanding population compared to a well-mixed population; for example, mutants that emerge right at the front can be carried along at the front and produce large clone sizes even in the absence of fitness benefits, in a phenomenon known as gene surfing (Hallatschek et al., 2007; Hallatschek and Nelson, 2008, 2010; Gralka et al., 2016; Farrell et al., 2017). In this work, we consider *de novo* diversity from a different perspective. While the clone size distribution considers the number of descendants arising from an individual mutation event, here we predict the total number of mutations (of any type) that are located at a particular spatial position within the biofilm. By tracking the lineages of microbes within the biofilm, we can predict patterns of *de novo* diversity, in terms of predicted mutation density, within the biofilm. However, since we do not connect the lineages of different microbes within the biofilm (i.e., we do not measure relatedness between individuals), we cannot track the fate of particular mutations. Therefore our work provides a different and complementary approach to understanding patterns of *de novo* diversity.

Computer simulations provide a powerful way to investigate phenomena that might be difficult to study experimentally, but they are not a substitute for experimental data. Tracking of lineages within experimental microbial populations is now possible, for well-mixed populations, using barcoding methods, although this has not been used for spatially structured populations (Blundell et al., 2019; Jasinska et al., 2020). For biofilms, advanced image analysis of growing biofilms allows the tracking of cell lineages in space and time (Jeckel and Drescher, 2021). Spatially-resolved detection of point mutations is challenging at present, but may well become possible in future. Therefore, experimental tests of the ideas presented in this work, although difficult, are not out of the question.

## Data availability statement

The raw data supporting the conclusions of this article will be made available by the authors, without undue reservation.

## Author contributions

EY and RA designed the study, interpreted the results, and edited the manuscript. EY performed the computer simulations, performed the data analysis, and wrote the manuscript. All authors contributed to the article and approved the submitted version.

## Funding

This work was funded by the European Research Council under consolidator grant 682237 EVOSTRUC and by an EPSRC DTA studentship awarded to EY. RA acknowledges additional support from the National Biofilms Innovation Centre (BBSRC BB/R012415/1). RA was also supported by the Excellence Cluster Balance of the Microverse (EXC 2051—Project-ID 390713860) funded by the Deutsche Forschungsgemeinschaft (DFG). For the purpose of open access, the author has applied a Creative Commons Attribution (CC BY) licence to any Author Accepted Manuscript version arising from this submission.

## Conflict of interest

The authors declare that the research was conducted in the absence of any commercial or financial relationships that could be construed as a potential conflict of interest.

## Publisher's note

All claims expressed in this article are solely those of the authors and do not necessarily represent those of their affiliated organizations, or those of the publisher, the editors and the reviewers. Any product that may be evaluated in this article, or claim that may be made by its manufacturer, is not guaranteed or endorsed by the publisher.
