# Supplementary material for: Lineage dynamics in growing biofilms: Spatial patterns of standing vs. de novo diversity
Source: Front Microbiol. 2022 Jul 27;13:915095. doi: 10.3389/fmicb.2022.915095 (PMC9363821; doi:10.3389/fmicb.2022.915095)
Supplement: Supplementary file 7 [file Data_Sheet_1.PDF]

Supplementary Material

1 SUPPLEMENTARY FIGURES

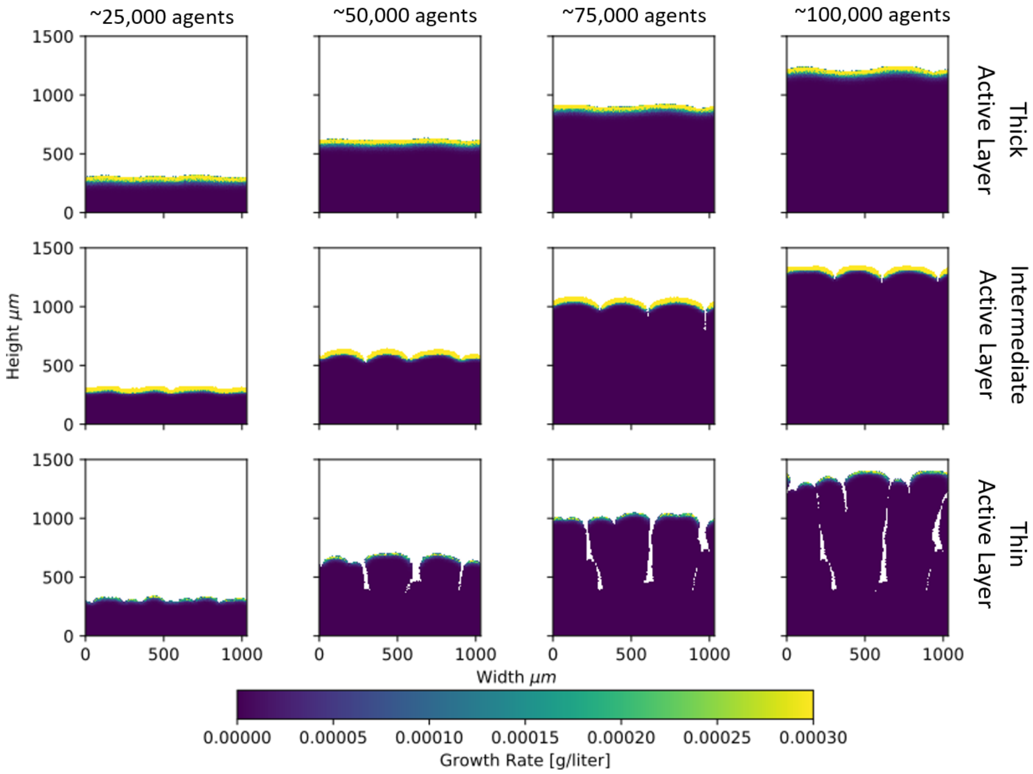

Figure S1. Biofilm morphology. Snapshots from our simulations of growing biofilms. From left to right, biofilm size increases from 25,000 to 100,000 agents. The colour coding show the local growth rate of microbes in the biofilm (see colour scale); growing cells are located in an active layer at the top of the biofilm (yellow region) while the majority of cells in the biofilm are not growing (dark blue region). The top, middle and bottom rows show simulations with different parameter values. The parameter values are as in the caption of Figure 1 in the main text. The average active layer thicknesses are: top row 102.8 ± 0.8 μm; middle row 71.30 ± 1.4 μm, bottom row 40.8 ± 1.4 μm. The morphology of the biofilm interface changes with active layer thickness; the simulation with a thick active layer has a smooth interface without gaps in the active layer (top row), the simulation with intermediate active layer thickness has a rougher interface with transient local gaps in the active layer (middle row), and the simulation with the thinnest active layer has gaps in the active layer that are permanent and lead to fingering of the interface (bottom row). See also the Supplementary Movies.

2 SUPPLEMENTARY TABLES

Average Active Layer thickness (μm)	μ_{max} (1/h)	S_{bulk} (g/liter)	Average agent specific growth rate (1/h)
29.86 ± 0.492	0.4	0.0005	0.0051
34.29 ± 0.99	0.4	0.001	0.0079
36.40 ± 0.90	0.3	0.0005	0.0040
40.79 ± 1.15	0.3	0.001	0.0071
44.91 ± 0.41	0.2	0.0005	0.0033
49.50 ± 1.37	0.4	0.005	0.0304
50.87 ± 1.03	0.2	0.001	0.0058
57.04 ± 1.18	0.3	0.005	0.0264
58.65 ± 1.25	0.4	0.01	0.0531
67.26 ± 0.99	0.1	0.0005	0.0025
68.47 ± 1.68	0.3	0.01	0.0456
71.30 ± 1.42	0.2	0.005	0.0217
74.60 ± 1.74	0.1	0.001	0.0043
84.4 ± 0.79	0.2	0.01	0.0364
102.79 ± 0.75	0.1	0.005	0.0144
125.43 ± 0.25	0.1	0.01	0.0232

Table S1. This table shows the average active layer thickness against the input parameters maximum specific growth rate of the cells μ_{max} and the bulk nutrient concentration S_{bulk}. The final column shows the average specific growth rate (μ) for microbes located within the active layer (see Fig. S6). All other simulation input parameters are kept constant and shown in Table 1.

3 SUPPLEMENTARY MOVIES

Movies showing the dynamics of loss of standing diversity corresponding to the three simulations show in Figure 2 of the main text. As in Figure 2, the shaded region indicates the active layer and the colours show the descendants of the 300 founder cells.

- Standing_diversity_thick_active_layer.mp4 (corresponds to top row of Figure 2)
- Standing_diversity_intermediate_active_layer.mp4 (corresponds to middle row of Figure 2)
- Standing_diversity_thin_active_layer.mp4 (corresponds to bottom row of Figure 2)

Movies showing the dynamics of lineage length corresponding to the three simulations show in Figure 5 of the main text. As in Figure 5, the shaded region indicates the active layer and the colours show the lineage length (colour scale as in legend of Figure 5).

- Lineage_Length_thick_active_layer.mp4 (corresponds to top row of Figure 5)
- Lineage_Length_intermediate_active_layer.mp4 (corresponds to middle row of Figure 5)
- Lineage_Length_thin_active_layer.mp4 (corresponds to bottom row of Figure 5)

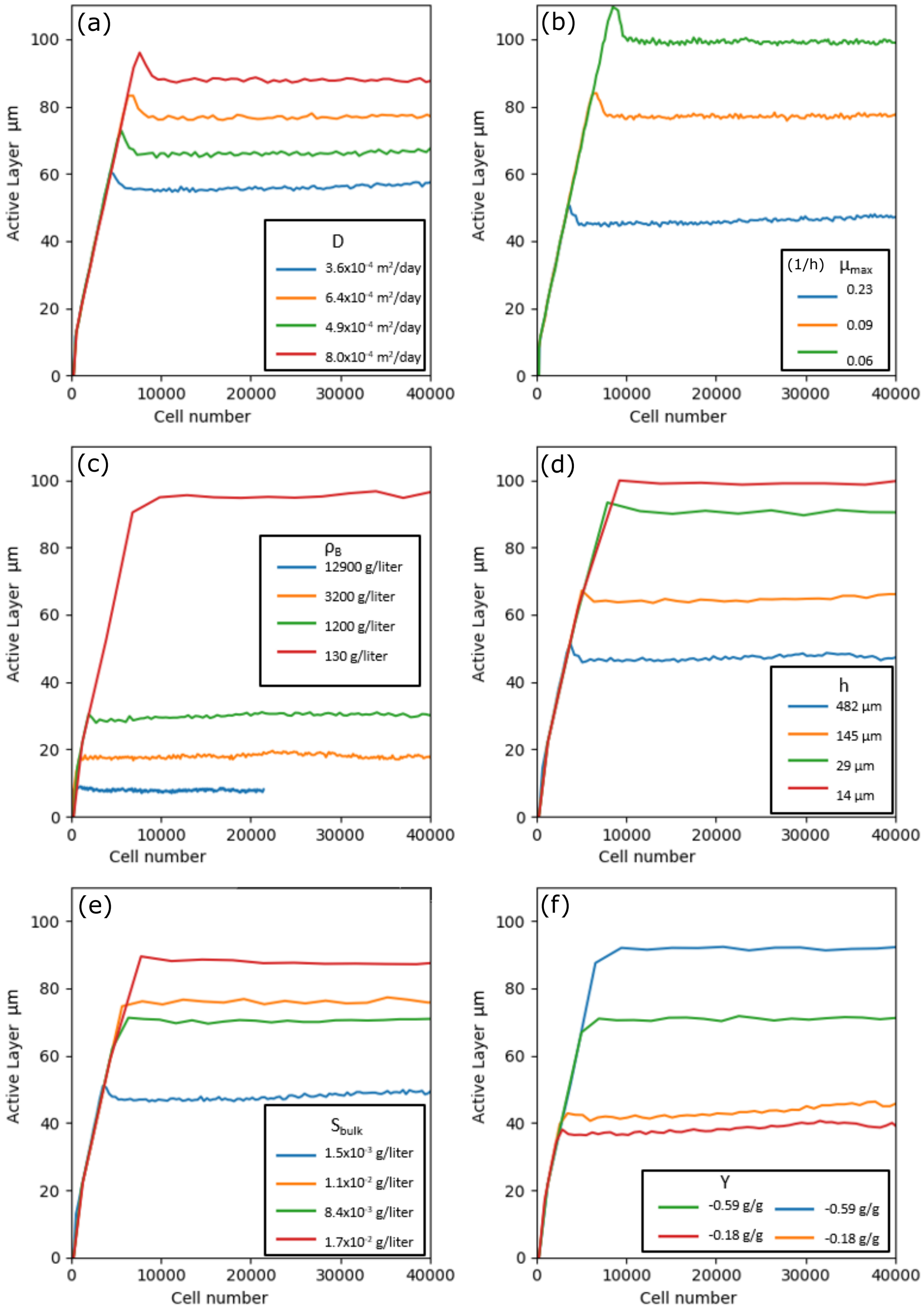

Figure S2. Average active layer thickness becomes constant in time. The active layer thickness, averaged across the biofilm interface, is plotted as a function of the biofilm size (cell number), a proxy for time. Each panel shows simulations where a different parameter is varied: diffusivity D in (a), maximum specific growth rate μ_{max} in (b), biomass density ρ_B in (c), diffusion boundary layer height h in (d), bulk nutrient concentration S_{bulk} in (e) and yield Y in (f) (see Table 1 of the main text). In all cases, the average active layer thickness quickly reaches a steady state. The process via which this steady state is reached can be qualitatively seen in the supplementary movies. In early biofilm growth, the nutrients penetrates all the way to the base of the biofilm, and the active layer thickness is equivalent to the thickness of the biofilm. As the biofilm grows, the nutrients can no longer penetrate all the way to the base of biofilm, and the active layer becomes a finite portion of the biofilm.

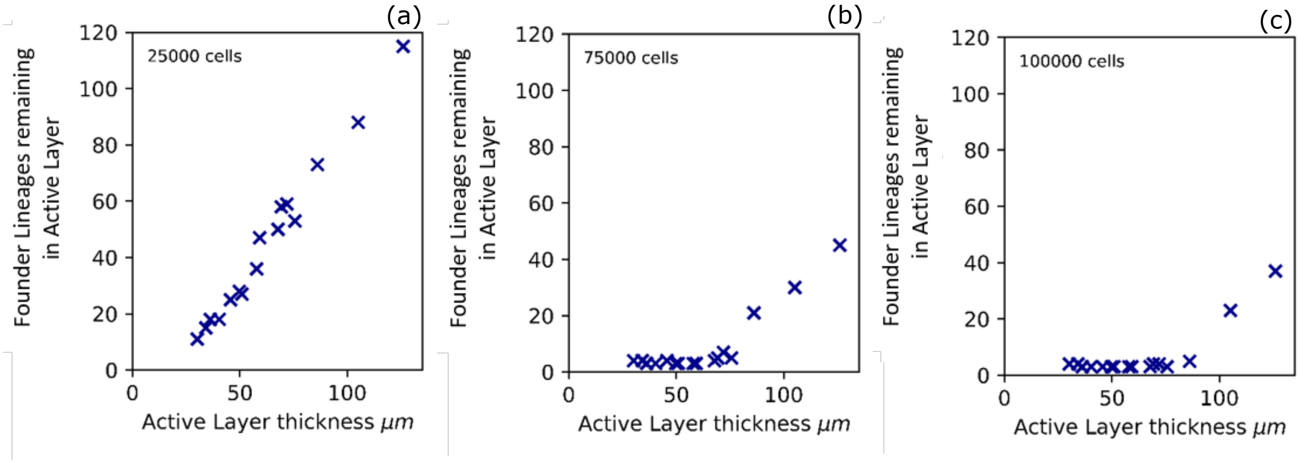

Figure S3. Active layer thickness controls loss of standing diversity. As in Figure 2 of the main text, we plot the number of founder lineages remaining in the active layer, as a function of the average active layer thickness, for a range of simulations with different parameter values (see Table S1). These plots are equivalent to Figure 2 of the main text but for different biofilm sizes (25,000, 75,000 and 100,000 microbes in sub-plots (a), (b) and (c) respectively).

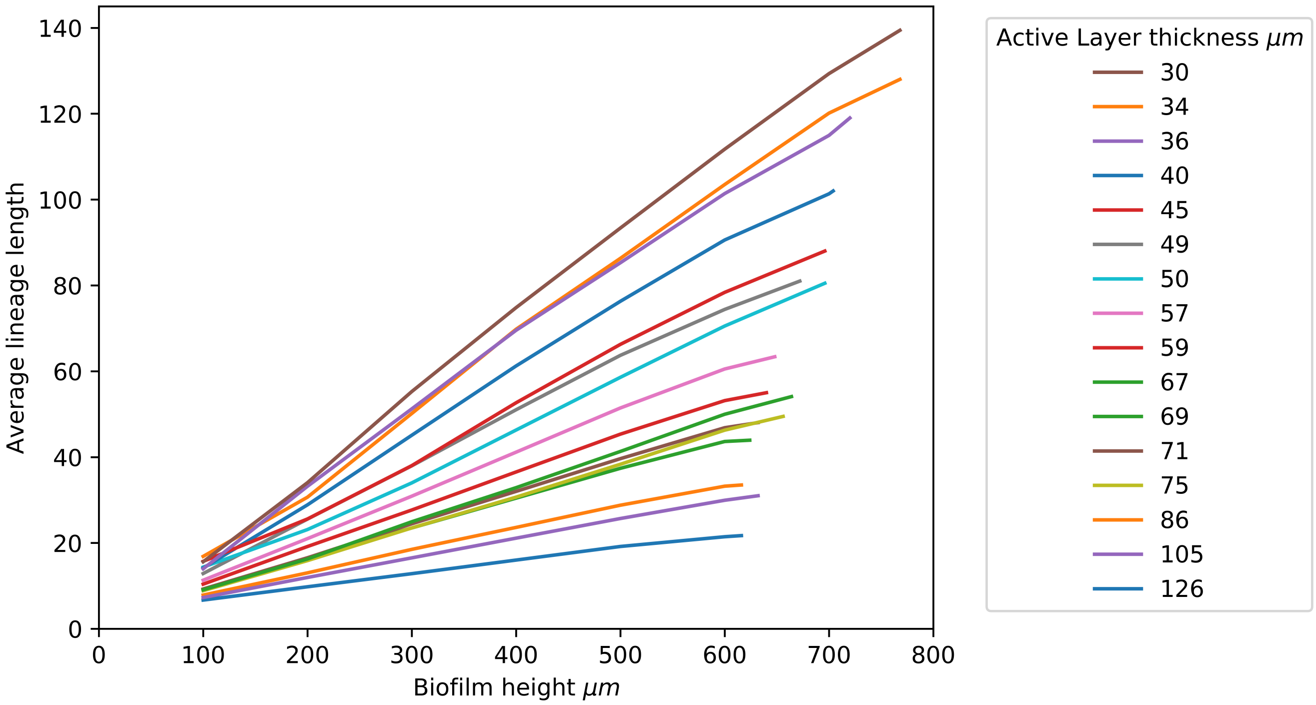

Figure S4. Spatial patterning of lineage length within the biofilm. For biofilms of size 50,000 microbes, lineage length is averaged for microbes at a particular height within the biofilm, and plotted as a function of height. The plot shows that the average lineage length of microbes within the biofilm increases linearly with height, such that microbes at the top of the biofilm have the longest lineages. Results are shown for a range of simulations with different active layer thicknesses (see legend); parameters are as in Table S1 and Table 1 of the main text.

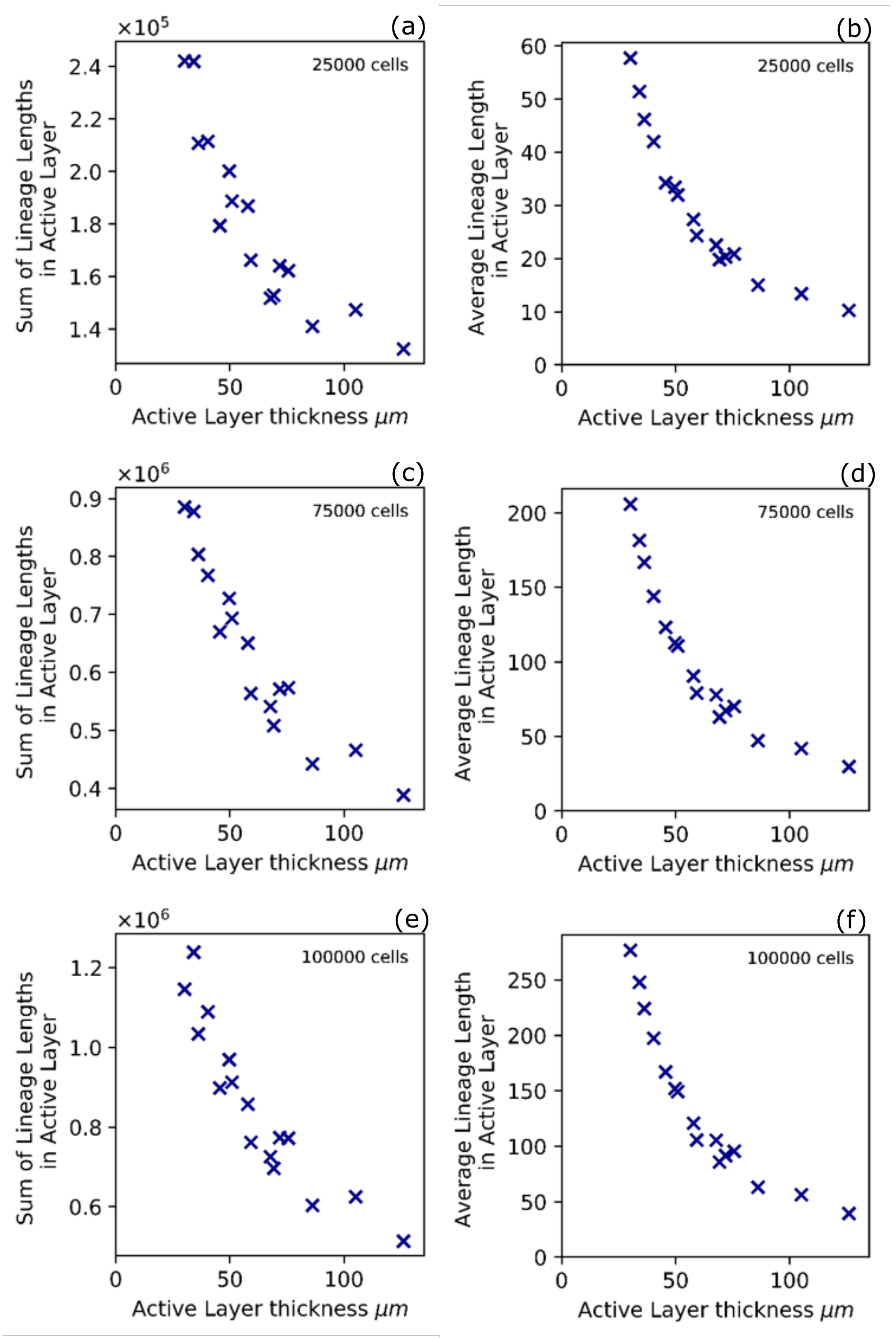

Figure S5. Active layer thickness controls patterns of lineage length. As in Figure 5 of the main text, we plot the sum of lineage lengths in the active layer (left plots i.e. (a), (c) and (e)), and the average lineage length in the active layer (right plots i.e. (b), (d) and (f)), as a function of the average active layer thickness, for a range of simulations with different parameter values (see Table S1). These plots are equivalent to Figure 5 of the main text but for different biofilm sizes (25,000 microbes in (a) and (b), 75,000 microbes in (c) and (d) and 100,000 microbes in (e) and (f)).

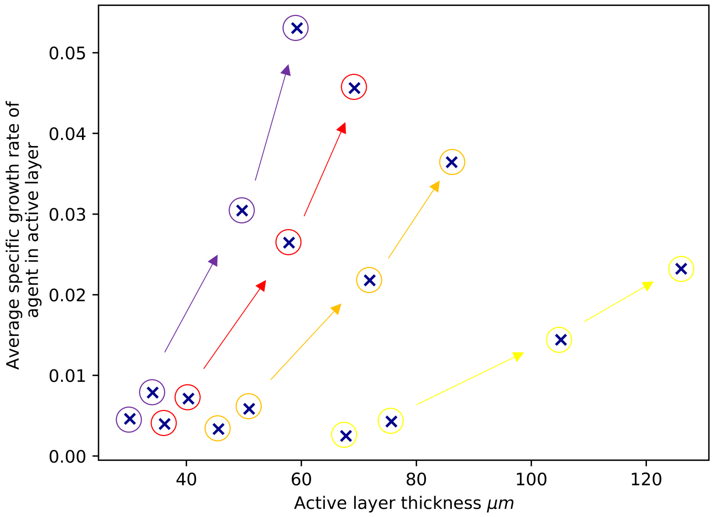

Figure S6. Active layer thickness correlates with average microbial activity in the active layer only when a single parameter is varied. For each set of parameters used in our simulations (i.e. the S_{bulk}, μ_{max} pairs listed in Table S1), the active layer thickness is plotted versus the average activity (specific growth rate) of microbes in the active layer. Each data point corresponds to a different parameter combination; the colours indicate the value of mu_{max} (purple = 0.4/h, red = 0.3/h, orange = 0.2/h, yellow = 0.1/h. The arrows indicate the direction of increasing S_{bulk}. If a single parameter is varied, the activity of microbes in the active layer correlates with the active layer thickness. However when we vary both parameters this correlation is lost. The data used in this plot is listed in Table S1.
